# The critical role of engineering in the rapid development of COVID-19 diagnostics: Lessons from the RADx Tech Test Verification Core

**DOI:** 10.1126/sciadv.ade4962

**Published:** 2023-04-07

**Authors:** Robert G. Mannino, Eric J. Nehl, Sarah Farmer, Amanda Foster Peagler, Maren C. Parsell, Viviana Claveria, David Ku, David S. Gottfried, Hang Chen, Wilbur A. Lam, Oliver Brand

**Affiliations:** ^1^ Department of Pediatrics, Emory University School of Medicine, Atlanta, GA 30322, USA.; ^2^ Behavioral, Social, and Health Education Sciences, Emory University, Atlanta, GA 30322, USA.; ^3^ Center for Advanced Communications Policy, Georgia Institute of Technology, Atlanta, GA 30332, USA.; ^4^ George W. Woodruff School of Mechanical Engineering, Georgia Institute of Technology, Atlanta, GA 30332, USA.; ^5^ Institute for Electronics and Nanotechnology, Georgia Institute of Technology, Atlanta, GA 30332, USA.; ^6^ Aflac Cancer and Blood Disorders Center, Children’s Healthcare of Atlanta, Atlanta, GA 30322, USA.; ^7^ Wallace H. Coulter Department of Biomedical Engineering, Emory University and Georgia Institute of Technology, Atlanta, GA 30332, USA.; ^8^ School of Electrical and Computer Engineering, Georgia Institute of Technology, Atlanta, GA 30332, USA.

## Abstract

Engineering plays a critical role in the development of medical devices, and this has been magnified since 2020 as severe acute respiratory syndrome coronavirus 2 swept over the globe. In response to the coronavirus disease 2019, the National Institutes of Health launched the Rapid Acceleration of Diagnostics (RADx) initiative to help meet the testing needs of the United States and effectively manage the pandemic. As the Engineering and Human Factors team for the RADx Tech Test Verification Core, we directly assessed more than 30 technologies that ultimately contributed to an increase of the country’s total testing capacity by 1.7 billion tests to date. In this review, we present central lessons learned from this “apples-to-apples” comparison of novel, rapidly developed diagnostic devices. Overall, the evaluation framework and lessons learned presented in this review may serve as a blueprint for engineers developing point-of-care diagnostics, leaving us better prepared to respond to the next global public health crisis rapidly and effectively.

## Teaser

Usability and scalability trumped technological sophistication during the rapid translation and deployment of COVID-19 tests.

## INTRODUCTION

The early days of the coronavirus disease 2019 (COVID-19) pandemic in the United States were characterized by rapidly increasing infections and hospitalizations that outpaced the ability of public health officials to confirm the diagnoses (*1*–*4*). Crucial efforts to trace contacts and confirm community transmission of COVID-19 were impossible without the ability to accurately diagnose infection by the severe acute respiratory syndrome coronavirus 2 (SARS-CoV-2) virus (*5*–*8*). Hence, diagnosing SARS-CoV-2 infection became a top priority for public health officials (*9*, *10*). To address this rapidly worsening public health crisis, the National Institutes of Health (NIH) launched an ambitious “Shark Tank”–style initiative in April 2020, in which biotechnology companies and biomedical researchers could pitch their ideas to rapidly bring new diagnostic technologies to market to meet the exponentially increasing testing needs of the population (*11*, *12*). As part of this Rapid Acceleration of Diagnostics (RADx) initiative, a wide variety of technologies at various technology readiness levels (TRLs) were submitted and funded, ranging from repurposed lateral flow assays (LFAs) originally designed for diagnosing other diseases or conditions to cutting-edge technologies using novel approaches to detecting respiratory infections (*13*).

**Fig. 1.**
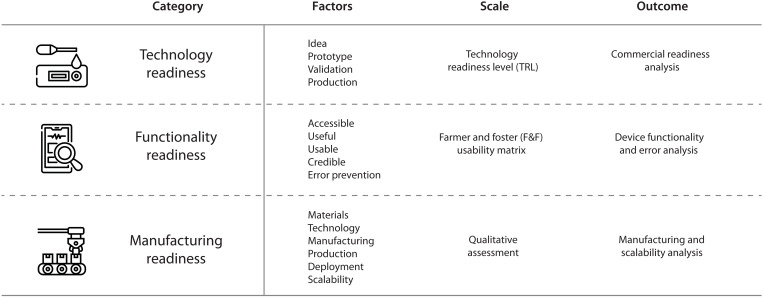
RADx engineering design and human factor readiness and maturity verification process. Our evaluations focused on the technology, functionality, and manufacturing readiness based on the scale, outcome, and a variety of factors for each parameter.

As the Engineering and Human Factors sub core of the RADx Tech Test Verification Core, we collaboratively evaluated more than 30 technologies that progressed through the tiered system of RADx funding (*14*). These comprehensive evaluations consisted of three principal phases. As can be seen from Fig. 1, we evaluated technology readiness and maturity using the TRL scale (*13*–*15*). In this phase, we assessed how far along the commercialization pathway each candidate technology was to clarify the timeline for market launch. Next, we assessed the functionality readiness of the device by performing tests and disassembly of the devices with limited prior instruction to analyze the underlying technology. This allowed us to approximate real-world use to assess usability, determine that the device was functioning as intended, and provide possible reasons as to why any errors or invalid tests may occur. Last, we evaluated the technologies from the perspective of manufacturability and scalability to ensure that the designs were optimized for commercial-scale production and to identify any barriers and provide possible corrective actions (*16*). Tightly coupled with the engineering analysis was a usability assessment by a team of engineers and scientists specializing in human factors (*14*, *17*). Note that the underlying serology test used to detect an infection, or any biomarkers used to establish a prognostic severity score, was not considered in our analysis. We focused on the form and function of the device itself.

Here, we present in detail our evaluation process of various approaches companies and research groups took for this historically productive period of diagnostic development and describe lessons learned from these observations and assessments. In particular, we focus on the techniques used in successful technologies that led to their availability for use by trained staff in clinical laboratories or for home use by patients themselves, as well as the mistakes that led to failure of some devices to reach commercialization. Given the unique and urgent circumstances presented by the COVID-19 pandemic, the analysis found here will provide insights into the field of successful diagnostic development. Considering the accelerated technology development timeline the pandemic demanded and the novel solutions the “Shark Tank” format of technology solicitation provided, insights gleaned from this review cover a broad range of issues surrounding biotechnology research and development. Overall, the evaluation framework and lessons learned presented here can serve as a blueprint to guide scientists, engineers, device developers, and funders to help mitigate the next global public health crisis.

## EVALUATION PROCESS

Many elements are required for a useful and commercially successful public health diagnostic tool for SARS-CoV-2. Device developers must balance usability with analytical performance while including features that allow for health authorities to monitor the spread and severity of the disease. This can be succinctly summed up with the REASSURED acronym, which was “coined to describe the ideal test to meet the needs of the developing world” in the context of improvements in digital technology in the diagnostic space (*18*). The ideal diagnostic device in a pandemic must (i) have real-time connectivity to allow public health agencies to monitor disease spread; (ii) have ease of specimen collection; (iii) be affordable; (iv) be sensitive and specific; (v) be user-friendly; (vi) be rapid and robust; (vii) be equipment-free, simple, and environmentally friendly; and (viii) be deliverable to end users. These qualities are important to consider when evaluating a diagnostic technology. The engineering evaluation process began with a careful review of company and technology documentation. After completion of this initial documentation review, any outstanding questions needed to clarify any perceived gaps in our understanding of the technology were directed toward the company either electronically or via a meeting. The engineering team then procured the technology, and on the basis of the technology incorporated within, the physical devices were distributed to the engineering team members with relevant expertise.

The devices were then analyzed by the team members by first performing the entire sequence of a test and then completing a comprehensive disassembly of the technology with evaluation of the individual components. If necessary, then experts in the field were brought in to evaluate specific aspects of the device. Each step in the process was thoroughly documented, and the notes and images collected were used to generate a detailed report of the analysis. The report opened with a TRL assessment to summarize the maturity of the technology (*15*). A detailed discussion of the functionality and breakdown of the technology components followed, where images and videos of individual components in action were taken and used as the source for this functional analysis. The results of the analysis were discussed in detail, and recommendations were made to correct design flaws and issues with functionality. This step considered results from the laboratory and clinical assessment of the technology, which was performed in parallel, and determined device sensitivity, specificity, limit of detection, and invalid rate (*14*). Last, we provided technology developers with an assessment of the manufacturability and scalability of the individual components of the technology and the overall device. These reports were passed along to the developers and the project leads at the NIH both to help the developers correct design flaws and issues with manufacturability/scalability and to assist the NIH with their funding decisions to help advance the technology.

In addition, usability evaluations were conducted to determine the viability of the intended use cases using a variety of methods, depending on the timeline of the project; the current stage of device development, physical access to the device; and the intended end users of the device. Evaluations were tailored to the system as a whole and took into consideration the limiting factors and circumstances of each project. These methods included expert reviews, design failure modes and effects analyses, task analyses, use case simulations, user observations, and user testing (*11*, *16*, *17*). Following evaluations, researchers provided actionable recommendations for increased usability. The recommendations focused on improvements to the protocol, the device, and/or the instructional materials and factored in the development stage of the device. Devices early in the design cycle received recommendations geared toward streamlining the protocol. For early-stage designs, physical changes to the device or major modifications to the protocol might be recommended to optimize user experience. For later-stage designs, emphasis was placed on smaller details and adjustments to instructional materials to reduce risk of error and enhance user satisfaction.

**Fig. 2.**
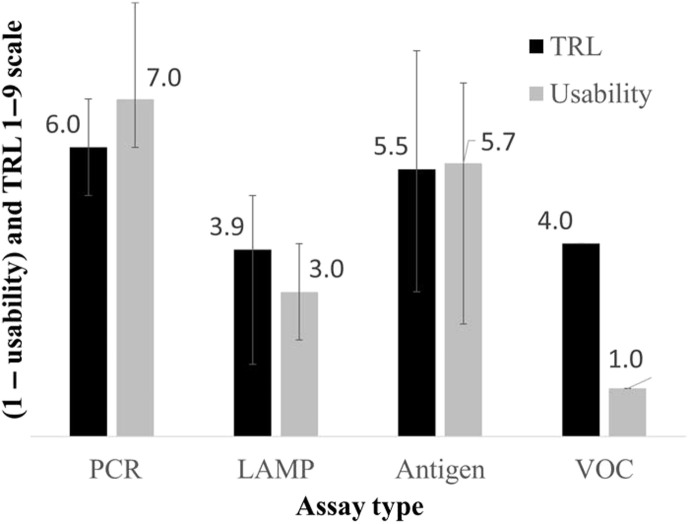
Average TRL and usability scores of evaluated technologies. Evaluated technologies were broadly broken down into the following categories based on the underlying virus detection method: polymerase chain reaction (PCR), loop-mediated isothermal amplification (LAMP), direct antigen detection, and volatile organic compound (VOC) analysis. Both usability scale and TRL are reported on a 1 to 9 scale. A lower usability score indicates as the most usable, and a higher TRL indicates as the most technical maturity. 1 – usability score was used to put highly usable and technologically mature technologies on the same scale.

Overall, we found that technologies using more established techniques for diagnostic testing [e.g., polymerase chain reaction (PCR)– and antigen-based tests] came to us as more mature and were easier to use (higher TRL and 1 – usability scores), while more experimental techniques were often less mature (Fig. 2). In this work, we present the summaries of our findings from conducting these evaluations and the lessons learned from them from the perspective of sample selection, technology utilization, and design (which includes functionality, usability, and scalable manufacturing).

## SAMPLE SELECTION, ACQUISITION, AND PROCESSING IN COVID-19 DIAGNOSTICS

**Fig. 3.**
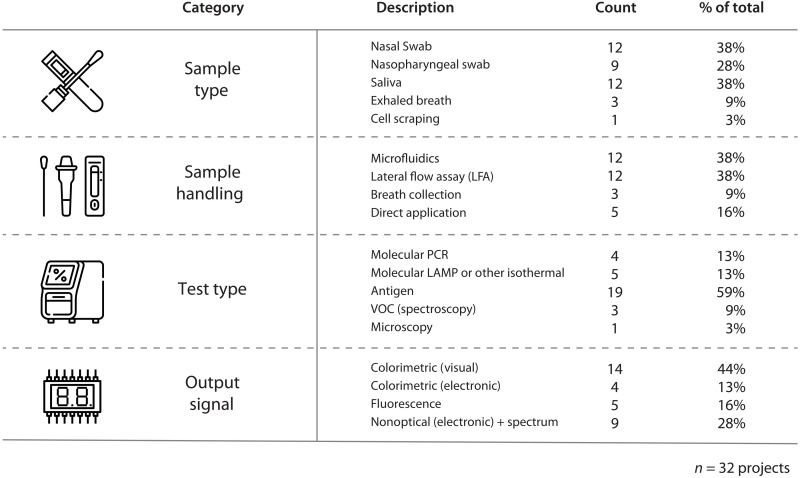
Technology breakdown of assessed RADx Tech projects. Note that some tests can use multiple sample types, so percentages do not add to 100%.

Sample collection is the first step in the testing process for COVID-19, and the collection method is typically the primary phase for patient interaction with the diagnostic device. Therefore, our evaluations began with an assessment of the sample collection procedure. In general, samples for SARS-CoV-2 virus rapid testing can be obtained from multiple sources, including swabs of the nasal cavity, saliva, blood, or exhaled breath, with nasal swabs and saliva being the most used by far (Fig. 3). Once collected, the sample was often mixed with various testing reagents and/or buffers, followed by multiple steps of mixing/vortexing and dilution to present a more homogeneous liquid sample to the detection device. Nasal samples were often taken from the nasopharyngeal (NP), mid-turbinate (MT), or anterior part of the nasal cavity (AN). NP swabs were among the first methods for collecting samples for SARS-CoV-2 and remain the preferred standard for the use of reverse transcription PCR (RT-PCR) and antigen diagnostic tests (*19*). Moreover, according to the U.S. Food and Drug Administration (FDA), NP specimens are generally considered to yield the most sensitive test results (*20*). However, NP swab samples are challenging to collect and need to be obtained by skilled technical staff, often causing patient discomfort during the sample collection. In contrast, MT swabs and AN swabs are substantially easier to collect (especially for self-collection) and are less invasive, making them more user-friendly methods for sample collection for rapid SARS-CoV-2 tests.

Swabs used for sample collection were typically made of synthetic fiber with plastic shafts, as outlined in the Centers for Disease Control and Prevention guidelines for the collection and handling of clinical specimens, for health care workers (*21*). Multiple specimens from the same patient may be taken with a single swab. If more than one swab was used for collecting specimens from two different locations in the same patient, then both swabs may be placed in the same vial to conserve collection and assay supplies. Calcium alginate swabs or swabs with wooden shafts should be avoided when testing for SARS-CoV-2 virus because they can contain substances that inactivate some viruses and can inhibit PCR testing (*21*). Swabs for SARS-CoV-2 virus testing can be readily found in the market, but for rapid SARS-CoV-2 virus rapid testing devices, some companies have developed their own custom-integrated swabs (which were included in our evaluations) for MT and AN sample collection as a part of their devices to optimize the sample collection process for their individual test (i.e., adjusting the length of the swab so that it fits both comfortably into the patient’s nose and snugly into the analysis area of the test).

Saliva sample collection was commonly used as an alternative to nasal swabs for SARS-CoV-2 detection (*22*–*25*). Saliva offers the advantages that its collection is easy, noninvasive, more acceptable for repeat tests, and can be performed by a non–health care professional or patients themselves with proper instruction. The typical collection methods include sponges inserted inside the mouth to absorb saliva, direct expectoration into a collection vial or cup, or a swab that collects saliva from different areas inside the mouth.

Several technologies were tested that used breath as a sample type. Each device generally used a mouthpiece where the patient could blow exhaled breath into the device. This mouthpiece typically contained a mechanism to prevent aerosolized virus from reaching the sensors within the analyzer that detected SARS-CoV-2 infection by measurement of unique nonviral analytes or metabolites rather than detecting the virus itself. These mechanisms were crucial to device usability because of the biosafety issue presented by device contamination. Different mechanisms for preventing device contamination were analyzed. One approach used a disposable bag to collect the breath sample, which was then transferred to a separate analyzer; therefore, the patient was never in direct contact with the reusable component of the test, i.e., the analyzer. Another approach saw the inclusion of a detachable, disposable mouthpiece that contained multiple filters and a desiccant bed to remove viral particles and humidity from the breath sample.

An additional, atypical, sample type tested was epithelial cells sloughed off from traditional swabbing of the interior of the mouth. These cells could then be analyzed via microscopy to determine the SARS-CoV-2 infection of individual cells. Theoretically, by imaging individual cells and analyzing for SARS-CoV-2 infection, a COVID-19 diagnosis could be made using a single infected cell.

Overall, our analysis of sample types and collection methodologies yielded several important lessons for scientists, engineers, device developers, and funders. Most importantly, the type of sample selected is critical for the specific assay. The invasiveness of the sample collection should be minimized while also maximizing the chances of obtaining high sensitivity given the range of viral loads that may be present in the given sample type. Furthermore, each sample type comes with an associated biosafety risk that must be recognized and minimized. In addition, we found that sample collection methods using swabs integrated into the testing device were generally easy to hold and maneuver. We found that the purpose of these integrated swabs was typically to give the user a point of reference for how far into the nasal cavity to insert the swab. This manufacturing feature is extremely helpful from a usability perspective for nonclinical end users, who lack experience working with standard commercial swabs. It was noted that, in some cases, the custom swab or parts of it (in case of swabs having a dedicated breaking point) containing the viral sample were fully contained by the vial or testing device, reducing the risk of cross contamination and simplifying sample/test disposal. We also found that successful technologies addressed the challenges of extracting biological material from the collection device. The sample type plays a role in the robustness of the material extraction solution, as different sample types are associated with different viscosity, composition, and quantity of biological material present (both target viral RNA and obstructing cellular material/mucus). Successful technologies typically addressed these challenges by using mechanical action squeezing or agitating the collection swab tip. Last, while saliva as sample matrix shows much promise because of the ease of collection, most point-of-care (POC) testing devices that obtained FDA Emergency Use Authorization (EUA) use nasal samples. It is believed that this is because of the complex, inhomogeneous nature of saliva, which makes it more difficult to be processed in typical tests and may need additional steps, such as filtering.

## TECHNOLOGY IN COVID-19 DIAGNOSTICS

Most of the POC technologies for COVID-19 detection supported by the RADx Tech program were either molecular or antigen tests. Molecular tests quantify SARS-CoV-2 viral RNA sequences using an amplification phase followed by transduction of the presence of specific RNA sequences into a qualitative or quantitative signal. Antigen tests use a sensing device to identify one or more SARS-CoV-2 antigens, such as the nucleocapsid (N) or spike (S) proteins.

Molecular tests and, in particular, assays based on real-time RT-PCR are the standard diagnostic tests used in centralized laboratories for the detection of COVID-19 (*26*, *27*). RT-PCR and other nucleic acid amplification tests are highly sensitive, but the handling in a centralized laboratory results in turnaround times of typically 1 to 3 days. RT-PCR tests are complex, multistep assays comprising cell lysis and RNA isolation, RNA amplification, and amplicon detection (*28*, *29*). Within the RADx program, we assessed several technologies that implement RT-PCR in a POC format with typical test turnaround times of 15 to 60 min depending on the technology. These POC technologies generally fall into one of two categories: (i) PCR tests using a disposable cartridge inserted into a (portable) instrument and (ii) PCR tests in a fully disposable format. For those devices with a disposable cartridge, we observed technologies that move the sample through a continuous microfluidic network to perform the necessary steps of the assay and technologies where a robotic fluid handling system embedded in the base instrument moves the sample between discrete reaction chambers embedded in the cartridge. PCR-based POC tests are highly complex with an intricate interplay of mechanical, fluidic, and electrical functionality, requiring pumps, valves, mixers, sensors, etc. While a self-contained, fully disposable device may be desirable for certain use cases, the waste generated by disposing the test with all its electrical, mechanical, and fluidic components is considerable even with recycling of components. In this respect, the cartridge-instrument approach typically is more sustainable and economically viable, with multiple tests performed at the same location in a short period of time. The complexity of the RT-PCR test requires excellent control and interplay of the different steps of the test to minimize invalid results. The test complexity also requires a lengthy research and development period, and PCR POC tests commercialized during the RADx program, such as the Thermo Fisher Scientific Accula (formerly Mesa Biotech) SARS-CoV-2 test, often had similar POC PCR products for other targets, such as flu, available or under development before the start of the NIH RADx program. RT loop-mediated isothermal amplification (RT-LAMP) assays detect viral nucleic acid using a constant temperature in contrast to the thermal cycling required for amplification in PCR (*30*). This reduces system complexity and, if combined with an LFA for visual readout, can potentially yield molecular tests for COVID-19 detection that only require a simple heater for operation, making it more suitable for resource-limited environments or home testing.

Antigen tests detect viral antigens by binding them to immobilized antibodies or other receptor elements on the test surface. Arguably, the simplest form of COVID-19 antigen tests is the paper-based LFAs with a visual readout, similar to common pregnancy tests (*31*). The sample is applied to an adsorbent sample pad, from where it flows through a conjugate release pad, where the target antigen binds to conjugated antibodies. The sample with conjugated antibody bound to the target antigen wicks along a paper test strip, which typically has two lines with immobilized biomolecules, a test line and a control line. Recognition of the target antigen results in a response at the test line, while the control line verifies proper liquid flow through the strip. The typical runtime of the LFA is 15 min. In an LFA with visual readout, the antibodies may be conjugated to gold nanoparticles, resulting in a visible color change (red) when the complex binds to control and/or test lines. The sample is ultimately collected by an absorbent pad at the end of the test strip. While the form factors of the LFA supported by the RADx program varied considerably, with some being simple test strips and others having the LFA housed in a plastic carrier, the quasi-standardization of the LFA and its components is considered a key advantage when it comes to (rapid) commercialization. Because of the maturity of the underlying technology, many of the POC COVID-19 tests supported by RADx that received EUA from the FDA and were introduced to the market during the program were actually LFA antigen tests. Their design, however, is not without challenges: The assay must have adequate sensitivity and selectivity to minimize false positives and false negatives and achieve consistent results from batch to batch. We sometimes witnessed that required sample volumes were increased to improve the test’s limit of detection; however, this might require a redesign of the test to be able to handle a larger sample volume without resulting in leakage, which is not tolerable with a potentially contagious sample.

LFA antigen tests are generally less sensitive than molecular tests, which include an amplification step, and low viral loads can result in faint test lines, which make the readout subjective to the ambient illumination and the individual reading subjective to the test result (*32*). Not only to minimize this subjectivity but also to improve the test’s limit of detection, several technologies combined the LFA with a sensor and readout, similar to digital pregnancy tests. In this case, the use of, e.g., fluorescent markers for conjugation combined with a suitable optical readout can improve the sensitivity of the test. An example is the Ellume COVID-19 Home Test. In addition, the resulting electrical test signal can be further processed, and the tests can interact with a smartphone-based app for reporting, aiding in public health efforts to track and manage cases but bringing its own security and data privacy challenges. In all cases, it is important to design the antigen tests with manufacturing in mind, i.e., ensuring that the components of an integrated analyzer or the reader can also be manufactured easily in high volume.

The LFA tests with separate readout devices first convert the viral antigen concentration into an optical signal, which is then converted using an optical sensor into a “processable” electrical signal. This poses the question of whether the viral antigen concentration could be directly converted into an electrical signal using a suitable sensor. In the literature, many such biosensors based on antigen-antibody interaction have been demonstrated in a laboratory environment for a variety of analytes (*33*, *34*). The idea behind all these sensors is that a suitable transducer detects target antigens bound to immobilized antibodies (or other receptors, such as aptamers) on the sensor surface or vice versa. Thereby, many different transducers can be used, with many examples using micro/nanofabrication technologies. In this context, we call them “chip-based” antigen tests because the transducer is often fabricated on a microchip. Similar to integrated circuits, these biosensors can be fabricated in high volume at low cost. Unfortunately, there were few such technologies that progressed through the RADx program, and among the few that we were able to evaluate, most did not achieve FDA authorization or commercialization success. One example that did achieve FDA EUA is the Qorvo Biotechnologies Omnia SARS-CoV-2 Antigen Test, which is based on an cartridge-instrument approach using a bulk acoustic wave resonator for detection. Why did not we see more chip-based antigen tests become successful? First and foremost, the time frame of the RADx program with an intended time to market of approximately 12 months certainly penalized complex and/or early-stage technologies. In the end, most of the technologies reaching EUA were based on proven, existing underlying technologies, such as LFA, with or without a reader, or even the conversion of established RT-PCR technologies into a POC format. But what can the biosensor community learn from the success of the LFA-based antigen tests? On one hand, having a biosensor, i.e., a chip that can detect an antigen (or more generally a biomolecule) in an ideal laboratory environment, is only part of a successful POC test. From the very beginning, developers must consider all test components, including sample delivery, processing, and routing; how to deal with common challenges, such as nonspecific binding, sensitivity variation, or signal drift; and ultimately how to manufacture the complete system, not only the chip, at high volume. To be ready for the next pandemic, we need more plug-and-play modules where novel chip technologies can be inserted into an existing platform, just as one can adjust the biochemistries in an LFA while the underlying platform (test strip, etc.) remains largely the same. Certainly, chip-based platforms for biomolecule detection are more complex, requiring fluid routing across the chip and featuring different (nonoptical) sensor signals, for example, so that more “standardized” platform technologies would benefit test development.

Devices use several methodologies for displaying results, which can be broadly categorized into analog or digital readouts. Analog readouts include changes in color, used in LFA-type antigen tests or in RT-LAMP assays with an LFA-type readout, displaying the results in terms of two-color outputs. Chemical reactions can also be linked to target-receptor binding, leading to color displays within microfluidic devices. Digital readouts were managed by interaction with an external device, tablet, or app, which performs results interpretation and gives the user a simple positive/negative result or in a device with all components integrated. Devices can automatically interpret on-board controls and display an “invalid” result if the control fails. These integrated readouts have the advantage of removing subjective user interpretation, and Bluetooth integration allows for at-home use that facilitates usability. Care must be taken to address issues of color blindness and legibility of result readouts to ensure that the end user can accurately identify the result of the test.

While many of the diagnostic devices that we analyzed used conventional test designs, we analyzed a few devices where nonconventional methods presented unique challenges, which we will highlight. The first nonconventional technology type used analysis of volatile organic compounds (VOCs) in breath samples. This technique required patients to breathe into a mouthpiece connected to a flow cell containing sensors for VOCs. This cell used ventilation to ensure that breath could enter the device, pass over the sensors, and exit the device without leading to pressure buildup. The fact that (potentially) virus-laden breath enters the reusable portion of the device turns the system into a potential fomite for viral infection, as the areas within the device that encounter breath can become contaminated by a COVID-19–positive individual and can infect a subsequent user. Further complicating this issue, we found that standard filtration methods designed to minimize this biosafety risk were less effective in filtering SARS-CoV-2 virus. The primary lesson learned from this experience for the safe use of reusable breath-based devices is to ensure that a closed loop between the patient and the reusable aspect of the technology is never formed. Moreover, because these breath tests do not detect viral components itself but a VOC signature stemming from a viral infection, careful signal analysis and interpretation are critical to minimize false positives and false negatives. This includes the analysis of conditions that could potentially lead to the presence of similar VOC patterns, such as other respiratory infections. Nevertheless, the simplicity associated with using breath as a sample medium to detect a SARS-CoV-2 infection is a strong argument for further exploring these technologies.

A second nontraditional approach used fluorescence in situ hybridization (FISH) to fluorescently tag viral RNA within an infected epithelial cell, which could then be imaged by a custom microscopy system. This system necessitated a microfluidic system to concentrate epithelial cells and present them for image analysis, while facilitating the FISH assay. We conducted a thorough microfluidics analysis that led to major design improvements in the device. Microfluidics should be optimized to minimize the presence of air bubbles forming within the channels. Air bubbles in the channels in this case caused impediments to fluid flow, which inhibited the ability of the fluorescent tag to consistently reach the infected cells, and this led to inconsistent image analysis and poor diagnostic sensitivity. The entire cycle of the sample once it enters the device should be considered. Liquid should not leak from the device and waste should be handled appropriately to ensure no biosafety issues arise.

## DEVICE DESIGN

### Manufacturing and scalability

In general, successful devices used robust, scalable, and standard manufacturing processes, including injection molding, punching, heat transfer components, and printed circuit board fabrication for many of its components. Alternatively, methods such as three-dimensional printing and soft molding are useful for prototyping purposes but are not sustainable and do not facilitate scalability. Device assembly is also a factor that must be considered. Assemblies that incorporate and require integration of different units and/or several different components may be an issue for rapid scale up of production. In this case, alignment of components for proper interaction can cause difficulties. Furthermore, a high number of components, some with moving parts, hinders the automation of assembly and limits the scalability. Tubing in microfluidic systems, involving the glued assembly of different fluidic components such as microfluidic chips, pumps, valves, fluidic chambers, and electrical wire connections between components, can also be problematic for device scalability. Daily manufacturing capacity at production scale should be considered. In this respect, the simpler and fewer components, the better. A good example of simple and effective device assembly would involve an LFA strip, optical components, and a printed circuit board (PCB) with electronic components, nicely aligned to each other with the help of simple pin-and-hole alignment features. In general, solutions such as the one used in this example were developed by companies that have used the technology for other applications that could be easily transferred to SARS-CoV-2 testing.

### Human factors and usability

An additional component of our engineering analysis was an assessment of human factors and usability. The primary tenet of human factors is to ensure that a system has been designed to be usable by the intended end user, with their various capabilities and limitations, in the intended environment of use. Before the COVID-19 pandemic, a shift from inpatient and laboratory diagnostics toward POC diagnostics was occurring, and the pandemic has only furthered this shift (*35*, *36*). With diagnostics occurring in nontraditional settings such as outpatient clinics, drive-through testing sites, and patients’ homes, considering the end user and the environment for use is more critical than ever. Under this paradigm shift, diagnostic test users vary more widely, as do their associated knowledge and skill sets. Users run the gamut from health care professionals conducting tests in outpatient settings to inexperienced volunteers conducting tests in community surveillance sites to individuals testing themselves in the home.

**Fig. 4.**
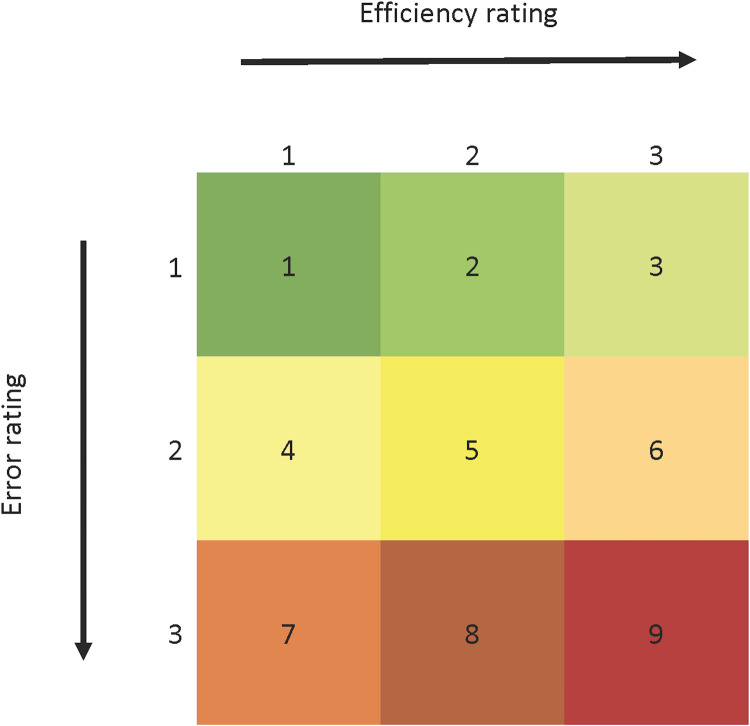
Sample F&F matrix for usability evaluation. This homegrown scale enables the assessment of new technology along two usability dimensions: error and efficiency.

Tests should be designed in such a way that the full range of potential users will have the capabilities and knowledge to successfully conduct the test. In addition, the entire system (the protocol, the device, and any instructional materials) was scored on a homegrown rating scale. The Farmer and Foster (F&F) usability matrix allowed us to score a system on a 3 × 3 matrix along two dimensions: efficiency and errors (Fig. 4) (*17*). The intention was to encapsulate as many components of usability as possible into the scale, with the caveat that access to the device and timeline were sometimes limited. This scale excludes some components of usability such as satisfaction (as user testing may not have been conducted) and effectiveness (these evaluations sometimes occurred before or in parallel to clinical validation). Inputs considered when scoring a system include the results from any human factor analyses conducted (e.g., expert reviews, heuristics evaluations, design failure modes and effects analyses, and root cause analyses), as well as feedback captured from users. While other groups have used alternate usability techniques such as Ishikawa diagrams to assess usability of COVID-19 diagnostics, the F&F rating scale was designed to provide us the ability to compare dissimilar tests in addition to the ability to communicate the system-level usability quickly and succinctly to the larger RADx team regardless of the evaluation techniques used (*37*, *38*). Scoring consists of the risk of error that would lead to system failure (error rating) and the expenditure of resources required from the user (efficiency rating). While this scale presents a high-level snapshot of usability only, it provides a consistent and efficient method by which to communicate the results of our human factor evaluations.

The range of testing environments offers its own set of challenges. Variable testing locations dictate that tests should be usable in a variety of settings—indoors, outdoors, and in temporary locations—and under a wide range of conditions, including various levels of humidity, temperature, lighting, and noise. While evaluating devices, three major considerations that could affect success or failure were identified as follows: process communication, accessibility, and system complexity (*17*). Process communication refers to the way in which the protocol is conveyed to the user. This could be through traditional instructional materials such as instructions for use documents or quick reference guides, as well as instructions displayed on the user interface, packaging, or on-device labeling. In general, a combination of these communication methods is preferred. Second, accessibility considers the user’s physical ability to complete the protocol in the intended environment. The design must consider how the users will be able to see, hear, and maneuver the components during the protocol. Reduced dexterity, visual acuity, and auditory acuity must be considered, whether because of the user’s individual ability (e.g., low vision or arthritis) or the environment (e.g., dim lighting or wearing gloves in cold weather). Designing for edge case scenarios, such as using large pull tabs for users with low dexterity, improves the accessibility for the target user group. Last, system complexity refers to the novelty and number of steps the protocol requires from the user. If a design requires the user to complete an action they have never encountered before, then the likelihood of error increases. Similarly, the user is more likely to slip up and encounter errors if they are required to complete a great number of steps. Requiring familiar actions, premeasuring solutions, and preassembled components are all ways to simplify the system and reduce the risk of error.

## FAILURE MODALITIES

**Fig. 5.**
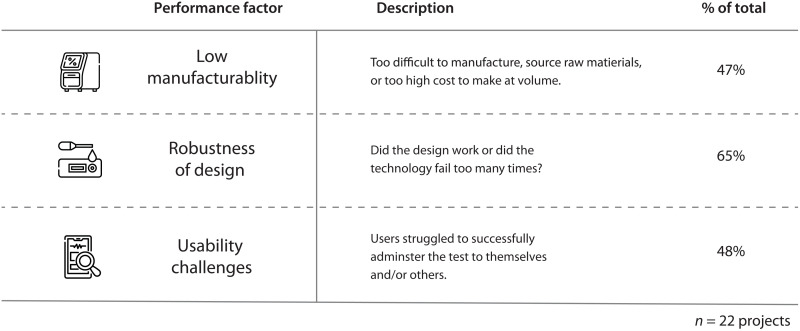
Failure modalities for evaluated technologies. Failure modalities could be generally categorized into three major groups: poor manufacturability, lack of robust design, and poor usability.

After analyzing more than 30 technologies, we found that the primary reasons for failure to achieve commercialization broke down into three main categories: manufacturability, robustness of design, and usability (Fig. 5). We found that most devices that failed suffered from robustness of design issues. These issues typically caused the devices to perform inconsistently, which ultimately lead to accuracy/diagnostic performance issues.

## CONCLUSION

The accelerated timeline of NIH’s RADx program, which targeted commercialization of COVID-19 testing technologies in a 6- to 12-month time, favored “established” technologies, such as antigen tests based on standard LFAs, or existing POC molecular testing platforms that could pivot to SARS-CoV-2 viral detection. Looking at the sample matrix, most POC tests supported under the RADx Tech program that received EUA use nasal samples. Multiple studies have demonstrated the usefulness of various nasal sample types for the detection of SARS-CoV-2 (*39*). Although saliva or breath might be easier to collect, sample complexity and inhomogeneity in the case of saliva and indirect detection of the viral infection in the case of breath sensing complicate the system design and have often resulted in performance degradation. In the case of saliva, proper sample preprocessing by, e.g., filtering appears to be a path forward. Looking at the output signal, most assessed testing technologies used optical techniques, not only most commonly colorimetric (either visual or electronic) but also fluorescence. Again, this seemed to be the conventional, proven approach. Nonoptical techniques and, especially, microchip-based electronic sensing technologies have, with few notable exceptions, proven difficult to develop and commercialize in the given time. It is believed that the large variety of possible nonoptical sensing technologies and their limited integration into full-sensing systems with proper sample handling and data processing has hindered their success within the program. More modular design concepts that allow for plug-and-play integration of these microchip-based technologies into standardized testing platforms are needed to advance these promising technologies. Last, manufacturing and human factors must be considered early on in the design process, as it is time-consuming and costly to address them later in the process. Ultimately, in “testing the tests,” a highly interdisciplinary team approach, with medical, engineering, human factors, and regulatory experts involved, has proven critical in providing meaningful feedback to the RADx Tech project teams and the funding agency.
